# Using Artificial Intelligence for Scholarly Writing

**DOI:** 10.1097/AJN.0000000000000179

**Published:** 2025-10-23

**Authors:** Marilyn H. Oermann, Jacqueline K. Owens, Heather Carter-Templeton, Gabriel Peterson, Hannah E. Bailey

**Affiliations:** **Marilyn H. Oermann** is Thelma M. Ingles Professor of Nursing at the Duke University School of Nursing, Durham, NC, and editor-in-chief, *Nurse Educator*. **Jacqueline K. Owens** is professor emerita at the Ashland University Schar College of Nursing and Health Sciences, Grafton, OH, and editor-in-chief, *OJIN: The Online Journal of Issues in Nursing*. **Heather Carter-Templeton** is associate professor at the West Virginia University School of Nursing, Morgantown, and editor, *CIN: Computers, Informatics, Nursing*. **Gabriel Peterson** is associate professor at the North Carolina Central University School of Library and Information Sciences, Durham. **Hannah E. Bailey** is a data analyst at Data Driven WV, West Virginia University, Morgantown. Contact author: Marilyn H. Oermann, marilyn.oermann@duke.edu. The authors have disclosed no potential conflicts of interest, financial or otherwise.

**Keywords:** AI bias, chatbots, generative artificial intelligence, scholarly communication, writing for publication

## Abstract

The widespread availability of generative artificial intelligence (genAI) continues to transform the scholarly communication process. With wide access to genAI tools, authors now not only have the benefits these tools can provide, such as creation of text, tables, and figures, but also the responsibility to use these tools with integrity and transparency. Examples of concerns about the use of genAI tools include ethical and legal breaches; inaccurate, biased, or fabricated content; and lack of accountability. Given the potential for serious harm to patients as well as the undermining of the credibility of scholarly communication with the use of unchecked content, it is essential for nurse authors to also include their judgment and subject matter expertise in the preparation of a scholarly manuscript that includes AI-generated information. This article offers a brief overview of recent research findings related to the use of genAI tools to support scholarly writing and provides guidelines for clinicians, educators, and other nurse authors on the appropriate use of AI in the preparation of manuscripts. Information is also provided about authorship, accuracy of content and references, biases and misrepresentations within AI-generated content, plagiarism, and appropriate disclosure of AI tools in manuscript preparation.

**Figure:**
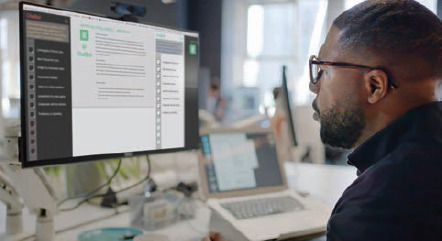
Photo by Laurence Dutton / iStock.

Scholarly communication is a process through which newly generated knowledge is disseminated to nurses and others for potential use in practice. Research findings and other forms of scholarship can be disseminated through formal methods such as articles published in peer-reviewed journals, which are critiqued by experts prior to publication and in many cases rigorously edited, and presentations at conferences. Informal communication methods, such as reports at meetings, online discussions, and social media, provide immediate and broad dissemination of information, although that information may not have been evaluated for its accuracy, lack of bias, or whether it is misleading.

**A process transformed**. The scholarly communication process has shifted over time with the wide availability of information that can be accessed online. Du and colleagues analyzed articles on scholarly communication from 1996 to 2021 to explore trends and areas of research.^1^ Their study revealed that social media and digitalization have transformed dissemination from research findings and other information only being shared through articles in peer-reviewed journals to broad and open dissemination using social media platforms, such as X (formerly Twitter) and Bluesky, and digital repositories, such as those available through universities, preprint servers like medRxiv (www.medrxiv.org), and data repositories such as the Center for Open Science (www.cos.io).

## OVERVIEW OF AI TYPES

Artificial intelligence (AI) is a broad category that encompasses many processes and tools. Specifically for scholarly communications, natural language processing, machine learning, deep learning, large language models, and generative AI (genAI) are the bases for the tools used most frequently and are subcategories of AI. Natural language processing includes various methods that use the rules of written language (computational linguistics) to recognize, analyze, and generate text along with recognizing tone and sentiment. Machine learning is a method of improving predictions on future data by training (and retraining) the models on known examples and data. Deep learning, which relies on a machine learning process, uses many layers of neural networks to train the predictive models on handling unstructured data—such as reports and pictures—so that the model will “think” more like a human brain would. The *large* in large language model refers to the data parameters: the different factors that affect the behavior of how the model is defined. There can be upward of over a trillion parameters with currently available technology. These parameters allow the large language models to generate human-understandable responses to prompt inputs.^2^ GenAI, as the name implies, *generates* new content that is like the content used to train it. This content can be text, images, music, and other forms. It should be noted that all these tools, methods, and models are only as good as the data used to train them.

## THE SURGE OF genAI USE IN SCHOLARLY COMMUNICATION

It is impossible for anyone with even minimal involvement with scholarly communication to miss the fact that a transformation of the process is occurring now with genAI tools, such as Chat Generative Pre-Trained Transformer (ChatGPT, OpenAI), Anthropic's Claude, and others. A survey in 2023 of more than 1,600 scientists revealed that 30% were using genAI tools to help prepare manuscripts.^3^ In 2024, a survey of approximately 3,000 researchers and health care professionals worldwide indicated that most (96%) had heard of AI, including genAI, and 54% had used it; importantly, 95% of the respondents believed that AI will have a significant impact on their work.^4^ Among all of the genAI tools, ChatGPT is the most well-known. This is not surprising. After ChatGPT was released to the public in November 2022, in only five days, it had reached over 1 million users. As of July of this year, ChatGPT.com was receiving more than 4.6 billion visits per month, with users sending 2.5 billion prompts daily.^5^

Authors are using genAI to search for information and create new content for manuscripts; develop outlines; draft, revise, and edit manuscripts; generate tables, figures, and illustrations; translate text into other languages; and for general administrative tasks. Many publishers, rightfully concerned about preserving the accuracy and integrity of the work they publish during this time of rapid change in authorship processes, have sought to define or limit the use of genAI by authors in certain ways. For example, most publishers will not allow images and illustrations that have been created using genAI. GenAI can produce images that appear real but are fake, potentially leading to image fraud with a serious impact on science and the scientific literature.^6^,^7^

Ng and colleagues surveyed more than 2,100 medical researchers in 2023 to assess their attitudes toward the use of AI chatbots such as ChatGPT.^8^ Benefits of AI-powered chatbots identified by respondents included time saved on drafting manuscripts, language translation, and creating figures and tables. More than half (61.8%) rated AI chatbots as very helpful or helpful for general administrative tasks. Examples of challenges when using AI for research included lack of information about how AI chatbots developed responses (77.2%); ethical and legal issues (76.8%); the inability of chatbots to reflect the “nuances and complexity” of researchers' perspectives and reasoning (76.4%); lack of accountability (73%); and privacy issues (72.9%).^8^ The use of AI chatbots has steadily increased in the two years since these findings, and researcher attitudes are likely to have shifted in some ways as well. But these researchers' concerns remain widespread and serve as a reminder that while genAI can perform many tasks associated with scientific writing, every manuscript needs humans who are experts in the topic to ensure accuracy and relevance of the content. The use of AI models, as they are refined and grow more powerful, will continue to develop in predictable and less predictable ways, but they cannot replace the expertise of clinicians, educators, and researchers.

## GUIDELINES FOR USING AI FOR SCHOLARLY WRITING

A recent study found that the majority of nursing journals did not have specific guidance related to the use of AI included in the information for authors.^9^ Among 258 nursing journals, 74.42% did not have any sections or instructions for authors in their author guidelines about using genAI or other types of AI tools in preparing manuscripts for submission to the journal. Only 27.13% of the journals referred authors to the publisher for this guidance.

Although the multiple uses of AI for scholarly writing are still evolving, there are some general guidelines that clinicians, educators, and nurse authors should follow when preparing a manuscript. This article provides the guidelines below on the appropriate use of AI tools in the preparation of manuscripts. These guidelines were developed based on a search of the literature from 2019 to 2025 in CINAHL Complete, PubMed, and Scopus; a review of recommendations from the International Committee of Medical Journal Editors, Committee on Publication Ethics, and World Association of Medical Editors; and guidance published in 2023 in *JAMA* on the use of AI in scholarly writing.

**Do not list an AI tool as one of the authors**. Based on International Committee of Medical Journal Editors standards, authors need to meet four criteria^10^:

- Make a substantial contribution to the work or the acquisition, analysis, or interpretation of the data.
- Participate in drafting a portion of the paper or in revising it.
- Approve the final manuscript.
- Be accountable for the accuracy and integrity of the work.

An AI tool cannot approve the final version of a paper (criterion 3) and cannot be held accountable for the accuracy and integrity of the content (criterion 4). With authorship comes accountability for the work, and none of the AI tools can assume this responsibility.^11^ A position statement about AI and authorship from the Committee on Publication Ethics and recommendations from the World Association of Medical Editors also add that AI tools cannot indicate conflicts of interest, which is required for submission of a manuscript, or agree to (and sign) copyright requirements.^12^,^13^

**Ensure content and references are accurate**. The author of a manuscript is responsible for the accuracy and relevance of the content produced by AI tools. When AI tools are used to generate content and to assist with writing and other tasks related to preparing the manuscript, the author is responsible for the integrity of the content.^14^ GenAI can quickly create content, tables, figures, and other documents for a manuscript, but the information it uses is derived from a wide range of sources on the Internet that are used for its training. These sources include peer-reviewed articles that are open access (freely available online to read), as well as other articles that are behind a paywall (require a subscription or willingness to pay for the article), but where publishers have authorized access to them for use in training large language models.^15^ In addition to articles that are peer-reviewed, books, proceedings, websites, blog posts, social media, and other information are also used for training, and this information is generally not first evaluated by experts. The information generated by AI tools may not be accurate and may not be relevant to the focus of the paper. The content derived from AI also may not be comprehensive because AI tools generate information based on their training and whatever was available on the Internet at that time.

It is well-known that AI tools may list citations (in the text) and references (in the list at the end of a paper) that are fake (often called “hallucinations”), may not be related to the topic of the paper, and may have errors in them. In a 2023 study by Walters and Wilder, slightly more than one-half (55%) of the ChatGPT-3.5 and 18% of the GPT-4 citations were fabricated; many of the actual (nonfabricated) references had errors in them (such as incorrect author names, volume, issue, or page numbers); and many had one or more American Psychological Association (APA) style errors, such as incorrect capitalization of the article title.^16^ In another study that evaluated the accuracy of citations and references in two different fields (sciences and humanities), of 102 citations, only 72.7% in the sciences and 76.6% in the humanities were real (actually existed in the literature).^17^ In addition, only 70.9% (sciences) and 74.5% (humanities) of the references listed were relevant to the topic of the paper. The digital object identifier (DOI), a string of numbers and letters that identifies a specific article or other document, was real in 70.9% of the references from the sciences but in only 38.3% of those in the humanities; the other DOIs were made up by ChatGPT.

Kacena and colleagues had ChatGPT write a scientific review article.^18^ Although AI reduced the time needed to write the article, the article required extensive review and time to confirm accuracy. Up to 70% of the references had errors. Concerns about inaccuracies and errors in references when using AI tools have also been discussed in nursing.^19^-^21^ A key implication is that authors should not use AI to obtain references.

**Check for biases and misrepresentations with AI-generated content**. Because AI tools are trained using diverse sources of information created by individuals and groups with potential biases, the content (text and images) generated using AI may contain biases based on race, gender, age, disabilities, and other areas. For example, an AI tool may create text and images that consistently depict the nurse as female and the physician as male. In one study, chatbots were two times more likely to predict Black people and Native American people as oncology nurses rather than oncologists, compared with Asian people.^22^ They also were more likely to predict women as oncology nurses rather than men. In another study in Australia, even though half of the paramedics are women, 100% (N = 32) of the images of paramedics and ambulance officers produced using genAI were portrayed as men, as White people, and as people with light skin tone.^23^

Fang and colleagues evaluated the gender and racial biases in content generated by seven different AI tools, including ChatGPT.^24^ They found that the content included significant gender and racial bias against women and Black people. It is important for authors using genAI for gathering information and preparing manuscripts to confirm that the AI-generated text and the language they use themselves in drafting the paper avoid bias.

**Ensure content generated from an AI tool is not plagiarized**. One of the concerns with using genAI is the issue of plagiarism. AI tools create content (and tables, figures, images, and other material) from previously published material used for training and may not specify their source.^13^ It is up to the author to establish that the material was not taken from a published article or other document. Before using any AI-generated content, authors should check the output to ensure it does not contain sentences or phrases from published text. One strategy to check for plagiarism in the AI text is to run the text through plagiarism checker software, which will scan the AI text for similarities against published content. Similar content can then be reviewed to determine if citations are needed. If the author does not have this software available in their setting, they can search online for a free plagiarism checker. The free versions, however, often have restrictions as to the number of words they search and may not be as accurate or thorough.

**Disclose how and where AI tools were used in preparing the manuscript**. Authors need to be transparent in their use of AI tools. While some AI-generated text may be apparent to editors and peer reviewers, it is often difficult to distinguish this from original text written by the author. In a study comparing abstracts published in journals with abstracts generated by ChatGPT, only a few of the ChatGPT abstracts were successfully identified.^25^ Without transparency, articles may be disseminating content produced using AI tools that is not accurate, among other concerns. Some studies have attempted to identify words and phrases that have appeared recently in articles and may indicate text that was created using AI.^26^-^28^ A study of the nursing literature found several nouns and verbs that increased significantly in articles starting in 2023, after AI tools became available to the public. For example, the word *intricate* increased by 435% in nursing articles and *delving* by 365%.^26^

When AI is used for producing drafts of text; revising content; and creating tables, figures, and illustrations for a manuscript, this needs to be disclosed in the manuscript, and the prompts and output should be provided in the text or as supplemental content.^10^,^13^,^20^,^29^,^30^ The author should state how the AI was used and where in the paper. The intent is to make the AI-generated content clear to editors, peer reviewers, and readers.

A statement can be added in an acknowledgment on the title page or prior to the references. The information about the genAI should include: the name of the AI software and version number, manufacturer, date it was used, and details about how AI was used and where in the manuscript.^31^,^32^ Guidance from JAMA Network also recommends that authors confirm that they assume responsibility for the content.^32^ See *Example of a Statement Disclosing Use of an AI Tool in a Manuscript*. Helpful resources for referencing AI in a manuscript include the *APA Style Blog* (https://apastyle.apa.org/blog/how-to-cite-chatgpt),^33^ *APA Publishing Policies*,^34^ and the *AMA Manual of Style*.^35^ Because many grammar-correcting software programs now have embedded AI to analyze text, correct grammar, and provide suggestions to improve writing, authors should also disclose when one of these programs was used in preparing the manuscript.

**Box 1:**
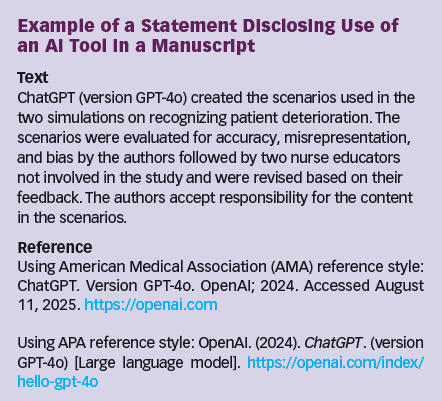
Example of a Statement Disclosing Use of an AI Tool in a Manuscript

## CONCLUSIONS

Since the appearance of AI tools in 2023, their adoption by researchers, clinicians, and authors has been rapid and is still growing. Little mystery exists about this enthusiastic embrace—AI tools make work easier. However, the limitations of AI (including lack of accountability and known problems with hallucinations and biased datasets) are also becoming better understood. Additionally, the internal logic of AI “reasoning” is often opaque to users. Unaddressed, these issues will begin to erode the quality and reliability of scientific literature, which is used to build evidence to guide clinical practices.

There are many uses of genAI for writing manuscripts about clinical studies and projects, but human expertise is essential to ensure the accuracy, relevance, and comprehensiveness of the content; that the content does not represent only one perspective and is not biased; that the text is not plagiarized and is attributed to the original authors; and that the references are actual publications, are relevant, and do not contain errors. Established author standards still apply when using AI; the use of new tools does not shift these responsibilities. Because articles may be used as evidence for clinical practice and as the basis of future research, it is important to disclose how and where AI tools were used through all phases of the project or study, and in preparing the manuscript. This is essential so the results are reproducible and can be used to build the evidence base for sound clinical practices.
